# Impacts of Symptom Checkers for Laypersons’ Self-diagnosis on Physicians in Primary Care: Scoping Review

**DOI:** 10.2196/39219

**Published:** 2023-05-29

**Authors:** Natalia Radionova, Eylem Ög, Anna-Jasmin Wetzel, Monika A Rieger, Christine Preiser

**Affiliations:** 1 Institute of Occupational Medicine Social Medicine and Health Services Research University Hospital Tuebingen Tuebingen Germany; 2 Institute for General Practice and Interprofessional Care University Hospital Tuebingen Tuebingen Germany; 3 Centre for Public Health and Health Services Research University Hospital Tuebingen Tuebingen Germany

**Keywords:** mobile health, mHealth, symptom checkers, artificial intelligence–based technology, AI-based technology, self-diagnosis, general practice, scoping review, mobile phone

## Abstract

**Background:**

Symptom checkers (SCs) for laypersons’ self-assessment and preliminary self-diagnosis are widely used by the public. Little is known about the impact of these tools on health care professionals (HCPs) in primary care and their work. This is relevant to understanding how technological changes might affect the working world and how this is linked to work-related psychosocial demands and resources for HCPs.

**Objective:**

This scoping review aimed to systematically explore the existing publications on the impacts of SCs on HCPs in primary care and to identify knowledge gaps.

**Methods:**

We used the Arksey and O’Malley framework. We based our search string on the participant, concept, and context scheme and searched PubMed (MEDLINE) and CINAHL in January and June 2021. We performed a reference search in August 2021 and a manual search in November 2021. We included publications of peer-reviewed journals that focused on artificial intelligence- or algorithm-based self-diagnosing apps and tools for laypersons and had primary care or nonclinical settings as a relevant context. The characteristics of these studies were described numerically. We used thematic analysis to identify core themes. We followed the PRISMA-ScR (Preferred Reporting Items for Systematic Reviews and Meta-Analyses extension for Scoping Reviews) checklist to report the study.

**Results:**

Of the 2729 publications identified through initial and follow-up database searches, 43 full texts were screened for eligibility, of which 9 were included. Further 8 publications were included through manual search. Two publications were excluded after receiving feedback in the peer-review process. Fifteen publications were included in the final sample, which comprised 5 (33%) commentaries or nonresearch publications, 3 (20%) literature reviews, and 7 (47%) research publications. The earliest publications stemmed from 2015. We identified 5 themes. The theme *finding prediagnosis* comprised the comparison between SCs and physicians. We identified the performance of the diagnosis and the relevance of human factors as topics. In the theme *layperson-technology relationship,* we identified potentials for laypersons’ empowerment and harm through SCs. Our analysis showed potential disruptions of the physician-patient relationship and uncontested roles of HCPs in the theme *(impacts on) physician-patient relationship.* In the theme *impacts on HCPs’ tasks,* we described the reduction or increase in HCPs’ workload. We identified potential transformations of HCPs’ work and impacts on the health care system in the theme *future role of SCs in health care.*

**Conclusions:**

The scoping review approach was suitable for this new field of research. The heterogeneity of technologies and wordings was challenging. We identified research gaps in the literature regarding the impact of artificial intelligence– or algorithm-based self-diagnosing apps or tools on the work of HCPs in primary care. Further empirical studies on HCPs’ lived experiences are needed, as the current literature depicts expectations rather than empirical findings.

## Introduction

### Background

Medical laypersons’ symptom self-assessment and deducing the necessity and urgency of contacting a physician is a core moment in medical care. Although self-diagnosis and self-triage in acute medical conditions or self-allocation to health care services beyond acute situations are not new, the internet has increased this phenomenon owing to the rapid and accessible health information that is available to consumers on the web. Web-based searches are a means for medical laypersons in general and for patients to inform themselves in this process. Representative studies in Germany have shown that 74% of laypersons use internet searches to inform themselves about health-related issues [1,2]. An older study from the United States showed similar results [3]. The negative effects of web-based searches on users have been described in the past 2 decades using terms such as *Dr. Google* [4] and *Cyberchondria* [5,6].

A novelty in this process is the plethora of artificial intelligence (AI)- or algorithm-based tools such as symptom checkers (SCs; Textbox 1 presents the definition). In addition to clinical decision support systems or diagnostic tools designed for physicians that directly impact their work [7], SCs are designed for laypersons. They provide laypersons with a preliminary diagnosis and suggest a course of action [8]. The field of SCs is heterogeneous with regard to technology, context, and specificity. Some SCs are browser–based, whereas others are (also) app–based [9]. Some SCs are embedded in the context of a specific health care system [10], whereas others operate globally [11]. Some SCs are tailored to specific diseases, such as COVID-19 [12,13], whereas others are more universal [11]. Some SCs not only suggest a course of action but also integrate further health-related functions such as symptom diaries. This heterogeneity poses challenges to research and practice.

**Relevant definitions.**

**Chatbots**

- “Chatbots, also known as conversational agents, interactive agents, virtual agents, virtual humans, or virtual assistants, are artificial intelligence programs designed to simulate human conversation via text or speech...In the context of health care, chatbots or *healthbots* are intended to provide personalized health and therapy information to patients, provide relevant products and services to patients, as well as suggest diagnoses and recommend treatments based on patient symptoms” [14].

**Symptom checker (apps)**

- “[Symptom checkers] generally provide people with possible alternative diagnoses based on their reported symptoms and/or suggest a course of action (eg, self-care, make a general practitioner (GP) appointment or go to an emergency department [ED])” [15].

From the perspective of occupational health, we are particularly interested in understanding how technological changes might affect the working world and how this is linked to work-related psychosocial demands and resources [16]. The Joint German Occupational Safety and Health Strategy derives 5 dimensions of work-related psychosocial factors from established models: “work content and task” (eg, completeness of tasks), “organization of work” (eg, working procedures), “social relations” (eg, social support from colleagues and managers), “working environment” (eg, workplace equipment), and “new forms of work” (eg, telework) [17]. These dimensions structured our perspectives on SCs in this study. In the United Kingdom, general practitioners (GPs) voiced concern that SCs might cause harm to patients, the physician-patient relationship, and the health care system in general [18]. In Germany, representatives of physicians’ associations have criticized plans for health insurance to implement an SC [19,20]. This can be understood as the first indication that some of these developments might affect the health care system and its professionals.

As SCs are a rather new technology, we expected limited knowledge and heterogeneous sources with regard to our research interests. Therefore, we chose to conduct a scoping review as a suitable method to gain an overview of existing knowledge [21]. Even though patients can access medical specialists directly in Germany, general practices are the first contact for patients [22] and will thus be the focus of our study. We will use the term health care professionals (HCPs) because we consider GPs and practice assistants alike. Even though diagnosis is the work content reserved for GPs as physicians, practice assistants tend to be the first contact persons for patients.

### Objective

This scoping review aimed to systematically explore the existing knowledge on the impacts of SC on HCPs in general practice and to identify gaps in knowledge. This review was guided by the following research question: *What is known from the literature about the impact of SC on HCPs in general practice, especially with regard to work content and work organization, HCP-patient interaction and relationship, professionals’ identity, job satisfaction, and perceived stress?*

## Methods

### Overview

A scoping review approach allowed us to identify, retrieve, and summarize publications relevant to a particular topic and to identify the key concepts underpinning a research area and the main sources and types of evidence available [23]. We chose a scoping review methodology, as it captures broad topics, different research aims, and study designs appropriately. We conducted our scoping review using the Arksey and O’Malley framework [23,24] and followed the PRISMA-ScR (Preferred Reporting Items for Systematic Reviews and Meta-Analyses extension for Scoping Reviews) reporting checklist for this publication (Multimedia Appendix 1).

Our scoping review included the following key phases [23]: (1) identifying the research question; (2) searching for relevant publications; (3) selecting publications based on predefined inclusion criteria; (4) extracting data; and (5) collating, summarizing, and reporting the results. We used an iterative review process, as we repeated all the main phases of all search strategies to include the latest publications. The scoping review was registered on an Open Science Framework platform on March 29, 2021 [25]; ethics approval was not required.

### Eligibility Criteria

We followed the population, concept, and context scheme [24] to define eligibility criteria. The relevant population included HCPs. We included publications with a focus on any patient or lay public facing AI- or algorithm-based SC technology (apps or similar tools) offering self-diagnosis, prediagnosis, initial diagnosis, self-anamnesis, or (initial) symptom assessment; prescreening or self-triage (concept); and a focus on general practice as a relevant context. The definitions are given in Textbox 1.

We included publications in peer-reviewed journals and did not restrict by study design to capture the wide array of relevant publications. The time frame from 2000 to 2021 was chosen to minimize the inclusion of irrelevant publications of older tools for self-diagnosis. The publications included were published in English, German, French, Turkish, or Russian and were obtainable in full text. We excluded studies that did not fit the topic and setting or focused on technologies that were designed for a professional target group such as tools for clinical decision support (Textboxes 2 and 3).

**Inclusion criteria.**

**Publication**

- Publication date from 2000 to 2021
- Languages: English, German, French, Turkish, and Russian
- All study types, views, editorials, etc

**Topic**

- Symptom checkers for general conditions

**Setting**

- General practice and primary care
- Health care professionals and physicians in general

**Digital technology**

- Used by lay public or patient-facing
- Artificial intelligence– or algorithm-based
- For self-diagnosis or self-triage

**Exclusion criteria.**

**Publication**

- For example, blogs, thesis, and dissertations

**Topic**

- Symptom checkers focused on specific medical conditions

**Setting**

- Hospital

**Digital technology**

- Used by physicians, nurses, etc
- Diagnostics with sensors, wearables, etc
- Clinical decision support systems, etc

### Search Strategy

As we expected limited results with regard to our specific research interests, we conceptualized a broad search strategy. We considered 2 medical databases, PubMed (MEDLINE) and CINAHL, as comprehensive databases for our research question and searched them systematically. We worked with a librarian at the University of Tübingen to construct a search term to ensure comprehensive results. We developed the search term using keywords and MeSH (Medical Subject Headings) terms from thematically relevant empirical studies [26,27]. Because the keywords for SC and self-diagnosing technology varied, we set out to include synonyms such as “chatbot” or “self-checker” to capture the heterogeneity of terms. As no current MeSH terms were closely related to our topic, we also used broader, related terms such as “smartphone” or “triage.” Initial scoping searches revealed that a highly sensitive search strategy including the population (HCPs) and context (general practice) demonstrated few search results and did not include known relevant publications related to SCs. We decided to use a broader search strategy to find more potential results and to be more specific when screening the search results. We adjusted our search strategy accordingly. It eventually consisted of three main concepts: (1) medical apps, (2) mobile health, and (3) (self-) diagnosis. The search query was then tailored to the specific requirements of CINAHL by a librarian. The final full search strategy for PubMed (MEDLINE) is presented in Multimedia Appendix 2.

The initial database search was conducted in January 2021, and the follow-up search was conducted in June 2021 to identify the publications published after the initial search. Subsequently, we reviewed the reference lists of the included publications and references of known relevant reviews on SCs [26,27] to identify further relevant publications in August 2021. We also searched manually for additional literature in relevant English and German journals (eg, *Journal of Medical Internet Research, European Journal of General Practice,* or *Deutsches Ärzteblatt*) and in Web of Science, ScienceDirect (June 2021), Google Scholar, and Google (November 2021) using keywords “symptom checker” and “self-diagnosis.”

### Publication Selection

We imported the database search results into the reference management system Citavi [28] and removed duplicates automatically or manually. We removed further publications because of their publication type, for example, commentaries and websites, and imported all citations (titles and abstracts) into the web app Rayyan (**Rayyan** Systems Inc) [29] to screen them for their relevance. The list was then screened by 2 reviewers (EÖ and NR) who decided the ratings of “include,” “exclude,” or “maybe” for each reference. The full publication was retrieved in cases of “yes” and “maybe” ratings when it was impossible to decide based on title and abstract. Three researchers (EÖ, NR, and CP) assessed the full-text publications for relevance, as abstracts might not accurately reflect whether a publication would fit our research interest.

The whole process (screening and selection) was conducted and constantly discussed by independent reviewers (NR and EÖ) to assess interrater reliability [30]. To ensure reliability between the 2 reviewers, we discussed methodological and content issues concerning publication screening [31]. A third reviewer (CP) was incorporated in situations of discrepancies to determine the final inclusion. We also discussed the challenges and uncertainties related to refining the search strategy with a fourth reviewer (MAR).

### Data Charting

We developed the data charting form following the Arksey and O’Malley framework [23]. NR and CP determined the variables to be extracted to answer the research question. The key information in the data was charted and managed using Microsoft Excel (Microsoft Corporation). Two reviewers (NR and CP) piloted the charting form with 4 publications to determine whether our approach to data extraction was consistent with the research question. We adjusted and finalized the charting form, and the same 2 reviewers (NR and CP) continually extracted data and updated the data charting form.

### Data Extraction

We extracted key information from the included publications pertaining to publication information (first author, year, type of publication, and country of origin); study information, if applicable (aim, setting, population data, sample size, methods, self-diagnosis tool types, and limitations); main findings; and implications related to our research question. All findings concerning the impact of SCs on GPs or HCPs were included. The main outcomes of interest were (impacts on) work content, organization or service use, job satisfaction of HCPs, perceived stress, HCP-patient or lay interaction or relationship and communication, and professional identity. Data extraction was performed simultaneously by 2 reviewers (NR and CP) and checked reciprocally for accuracy and consistency.

### Synthesis of Evidence

We used thematic analysis [32] to structure the key considerations around the research questions and to outline the knowledge gaps in the literature. Two researchers (EÖ and NR) familiarized themselves with the extracted qualitative data by repeatedly reading the data to enhance their understanding and conducted the analysis independently to identify common patterns and themes. The data were thematically coded (EÖ and NR) and categorized into meaningful themes and subthemes (CP and MAR) [32]. The results of the analysis were discussed by the research team (EÖ, NR, CP, and MAR) to find an agreement for the final set of findings. The themes were then validated by 2 researchers (NR and CP), and methodological questions were discussed with a research partner from the research network CHECK.APP (AJW) [33], with which this work is associated but not funded.

## Results

### Characteristics of Sources of Evidence

**Figure 1:**
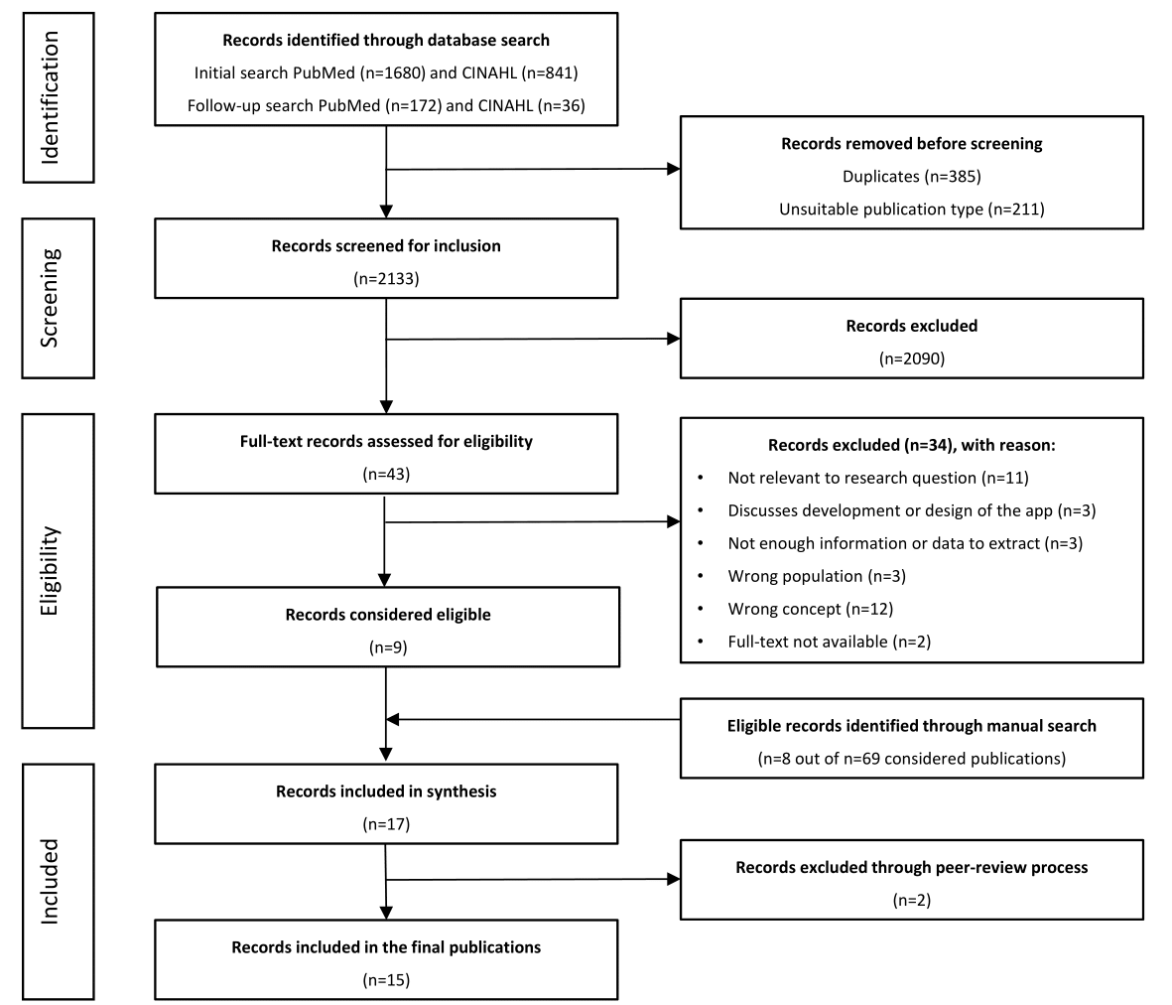
PRISMA (Preferred Reporting Item for Systematic Reviews and Meta-Analyses) flowchart of the study selection process.

A numerical analysis of the selection process and the included publications is presented. Of the 2729 publications found in the database search, 385 (14.11%) duplicates and an additional 211 (7.73%) publications with unsuitable publication types were removed. Of the remaining 78.16% (2133/2729) of publications, 2090 (97.98%) articles were removed after screening for inclusion, and 43 (2.02%) articles were screened for eligibility, of which 9 (21%) publications were considered eligible. A total of 69 publications were included through manual search and screened for eligibility, which resulted in further 8 (12%) included publications. The number of included publications for data extraction was 17. On the basis of the detailed feedback during the peer-review process in this journal, we excluded 2 more publications. The final number of included publications was 15 (Figure 1).

Most publications (5/15, 33%) were published in 2019 [14,15,34-36]. The oldest publications (3/15, 20%) concerning our research questions were published in 2015 [37-39], whereas the most recent publications (4/15, 27%) were published in 2020 [40-43]. Of the 15 publications included in this scoping review, 8 (53%) were related to SCs, self-triage services or tools or apps, and web-based self-diagnosis [15,37-41,43,44]; 5 (33%) were related to chatbots [14,35,42,45,46]; and 2 (13%) were related to AI in diagnosis [34,36]. We distinguished between 33% (5/15) of commentaries or nonresearch publications, 20% (3/15) of literature reviews, and 47% (7/15) of research publications. The commentaries as nonresearch publications included editorials [36], position papers, or overviews of technologies [35,44-46]. We found there 20% (3/15) of studies used a qualitative approach [34,38,39], 20% (3/15) of studies used a quantitative approach [14,37,42], and 7% (1/15) used a mixed methods approach [41]. Although 47% (7/15) of publications were about the primary care setting [14,34,36,40-43], the setting was not specified in 53% (8/15) of publications [15,35,37-39,44-46].

### Results of Individual Sources of Evidence

We identified 5 themes in the heterogeneity of the included publications. Table 1 provides an overview of the main themes.

The charting table (Table 2) provides an overview of the included publications and outlines the following variables: first author, setting in health care, publication type and study type, information about the AI-based technology type, and the overview of the identified main themes concerning our research questions.

**Table 1:** Symptom checkers (SCs) and health care professionals (HCPs): overview of main themes in the publications included.

Theme, topic, and subtopic				Research		Literature review		Nonresearch
**Finding prediagnosis: comparison between SCs and physicians**								
	**Performance of diagnosis**							
		Lower accuracy of SCs in diagnostics and triage	[14,37,38]		[15,40,43]		N/A^a^	
		Physicians consider various sources of information	N/A		N/A		[37]	
		SCs provide a first sense of urgency	[37-39]		N/A		N/A	
	**Relevance of the human factor**							
		SCs lack human factor	[39]		N/A		[36,44,46]	
		Human factor as a strength of physicians	[34]		[43]		N/A	
**Layperson-technology relationship**								
	**SCs as potential for layperson empowerment**							
		SCs might provide laypersons with knowledge	N/A		N/A		[45]	
		SCs might have layperson activating potential	N/A		N/A		[46]	
	**SCs as potential harm for laypersons**							
		SCs' quality and liability still unclear	[42]		N/A		N/A	
		SCs might increase anxieties in laypersons	N/A		N/A		[45]	
**(Impacts on) physician-patient relationship**								
	**Disruptions of the physician-patient relationship**							
		Potential of changing roles	[38,39,41]		N/A		[35]	
	**Uncontested role of HCPs**							
		Improvement of relationship	N/A		N/A		[46]	
		HCPs remain the primary source of trustworthy information	[39,42]		N/A		N/A	
**Impacts on HCPs’ tasks**								
	**SCs reducing HCPs’ workload**							
		SCs could support repetitive and less-complicated tasks	[14,34]		N/A		[35]	
		SCs could enable physicians to concentrate on other tasks	[34]		N/A		[35,45]	
	**SCs increasing HCPs’ workload**							
		SCs might cause unnecessary contacts	[41]		[40]		[36]	
		SCs might cause additional tasks during consultation	[41]		N/A		[45]	
**Future roles of SCs in health care**								
	**SCs transforming the work of HCPs**							
		SCs transform the roles of HCPs	[34]		N/A		N/A	
		SCs complement HCPs	[14,34,41]		[43]		[37]	
		SCs in competition with HCPs	[14]		[43]		N/A	
	**SCs' impacts on the health care system**							
		Improved supply for patients	[41]		N/A		[45]	
		Potential overuse of service through patients	[37]		N/A		N/A	

^a^N/A: not applicable.

**Table 2:** Charting table of included publications.

Publication details					Main themes				
First author, year, reference	Setting in health care	Type of publication and study type^a^	SC^b^ or AI^c^-based technology^d^	Finding prediagnosis: comparison SCs and HCPs^e^		Patient-technology-relationship	(Impacts on) physician-patient relationship	Impact on HCPs’ tasks	(Future) role of SCs in health care
Blease et al [34] (2019)	Primary care	Research: qualitative approach	AI technology	✓		N/A^f^	N/A	✓	✓
Chambers et al [15] (2019)	Setting not specified	Literature review	SC and triage services	✓		N/A	N/A	N/A	N/A
Denecke et al [35] (2019)	Setting not specified	Nonresearch: commentary	Chatbots or intelligent conversational agents	N/A		✓	✓	✓	N/A
Gottliebsen and Petersson [40] (2020)	Primary care	Literature review	Digital tools with triage service	✓		N/A	N/A	N/A	N/A
Jutel et al [38] (2015)	Setting not specified	Research: qualitative approach	Medical diagnosis smartphone apps	✓		N/A	✓	N/A	N/A
Kuhn et al [45] (2018)	Setting not specified	Nonresearch: commentary	Chatbots	N/A		✓	N/A	✓	✓
Kujala et al [41] (2020)	Primary care	Research: mixed methods approach	Web-based SC	N/A		N/A	✓	✓	✓
Lupton et al [39] (2015)	Setting not specified	Research: qualitative approach	Self-diagnosis smartphone apps or SC	✓		N/A	✓	N/A	N/A
Merz et al [44] (2018)	Setting not specified	Nonresearch: commentary	(Self-)diagnosis apps	✓		N/A	N/A	N/A	N/A
Miles et al [42] (2020)	Primary care	Research: quantitative approach	Chatbots (for diagnosis and triage)	N/A		✓	✓	N/A	N/A
Müschenich et al [46] (2018)	Setting not specified	Nonresearch: commentary	Chatbots	✓		✓	✓	N/A	N/A
Palanica et al [14] (2019)	Primary care	Research: quantitative approach	Health care chatbots	✓		N/A	N/A	✓	✓
Semigran et al [37] (2015)	Setting not specified	Research: quantitative approach	SC	✓		N/A	N/A	N/A	✓
Summerton and Cansdale [36] (2019)	Primary care	Nonresearch: commentary	AI-based diagnosis	✓		N/A	N/A	✓	✓
Wattanapisit et al [43] (2020)	Primary care	Literature review	Mobile health apps for self-diagnosis	✓		N/A	N/A	N/A	✓

^a^If applicable.

^b^SC: symptom checker.

^c^AI: artificial intelligence.

^d^Tools or technology concerning research questions and eligibility criteria.

^e^HCP: health care professional.

^f^N/A: not applicable.

### Synthesis of Results

#### Finding Prediagnosis: Comparison Between SCs and Physicians

One theme that repeatedly occurred in the publications can be grouped under the *performance of diagnosis* and the *relevance of the human factor*. We see the former as part of the work content and the latter as the basis for the quality of performing the work content.

##### Performance of Diagnosis

Some authors attested that SCs have a *lower accuracy in diagnostics* [14,15,37,40,43] and in *triage,* understood as an evaluation of urgency [15,36,37,40], compared with physicians. The authors stated that SCs lack situational [38], holistic, detailed [14], or individualized [34,38] knowledge of patients. Some authors emphasized the complexity of physicians’ diagnostic work as *they consider various sources of information* and contextual factors [36]. Some authors limited the expectations toward SCs. In their view, SCs can provide the user with *a first sense of urgency* [38] and a possible diagnosis [37]. Furthermore, SCs were not the only diagnostic method [39].

##### Relevance of the Human Factor

According to some authors, SCs *lack a human factor*. SCs do not have “tacit knowledge such as how to gain an individual’s confidence” [36]. The lack of human factor was framed more positively by some authors: SCs have the potential to be more neutral and objective [39] as well as to be evidence based, faster, more accurate, error free [39,44], and less biased than GPs [36]. Unlike physicians, SCs “do not get tired or irritable” [36] and provide 24-hour accessibility [46]. Some authors viewed the *human factor as a strength of physicians* to whom they ascribed a “6th sense” and “respectful,” individualized care [34] as well as holding “integrative skills, art, values, and ethics” [43].

#### Layperson-Technology Relationship

Some authors addressed the impacts of SCs on layperson users as (potential) patients. We consider this relevant to our research question, as it might indirectly transform HCPs’ work content.

##### SC as Potential for Laypersons’ Empowerment

Authors saw the potential of SCs to *provide laypersons with knowledge*, as they might function as a better alternative concerning health-related issues, for example, in comparison with Google and Wikipedia [45]. Some addressed the *layperson activating potential of SCs*, as laypersons might switch into a more active role through self-management and responsibility [46].

##### SC as Potential Harm for Laypersons

According to some authors, SCs could be potentially harmful for laypersons, as the *issues of quality and liability are still unclear* [42]. The authors voiced concern that the use of SCs *might increase anxieties in laypersons* [45].

#### (Impacts on) Physician-Patient Relationship

Some authors addressed the impacts of SCs on the physician-patient relationship. We consider this to be relevant because the physician-patient relationship is also part of the HCPs’ work content (whereas the relationship between practice team members is part of social relationships).

##### Disruptions of the Physician-Patient Relationship

Some authors addressed the *potential of changing roles* in the physician-patient relationship. The authors depicted the patient-physician relationship as relying on trust and face-to-face conversation [35], which is also structured through a power imbalance between laypersons and physicians [38]. SCs could cause an “uneasy space between the engaged patient and the expert medical professional” [39]. The amplified role of laypersons in the diagnostic process and prediagnostic work might lead to the redistribution of power in the physician-patient relationship [38] and challenge professional dominance, medical authority, and expertise [38,39,41].

##### Uncontested Role of HCPs

Some authors indicated that SCs might have an impact but that the roles of HCPs would remain unchallenged. Some authors voiced that SC might have *positive effects* on the physician-patient relationship. SCs can enable and intensify necessary physician-patient contact [46]. According to some authors, physicians *remain the primary source of trustworthy information*; GPs were favored over technology, especially in conditions perceived as highly severe [42]. In addition, the HCPs’ credibility, competence, and the art of encouragement were not questioned, and the internet was seen as a complementary, not a competing, source of information [39].

#### Impact on HCPs’ Tasks

The impacts of SCs on HCPs’ tasks is another theme in the literature relevant to our research question, as it is linked to work content. We included those parts that might have an impact on HCPs’ work.

##### SCs Reducing HCPs’ Workload

In some publications, SCs were seen as reducing HCPs’ workload through providing or *supporting repetitive and less-complicated tasks* for the HCPs such as “initial triage of uncomplicated patients” [34], “self anamnesis” [35], “patient education” [35], and “administrative and organizational tasks” [14]. This could *enable physicians to concentrate on other tasks* such as verification and interpretation of the provided information [35] and to focus on “proper diagnosing and doctoring” [34]. Generally, it could support the consultation [35,45].

##### SCs Increasing HCPs’ Workload

Some authors assumed that SCs might increase HCPs’ workload. *Unnecessary contacts* might increase [40] as patients might overcontact HCPs through several channels [41] or as SCs might “encourage users to contact GPs even though self-care would be reasonable and safe” [36]. The workload might also increase through *additional tasks during consultation*. HCPs might need to explain the conflicting results between their assessments and those of SCs [41]. HCPs might need to acquire new knowledge to understand the technology used by patients [45].

#### Future Role of SCs in Health Care

Some authors made prognoses for the future of SCs and their impacts on health care. We consider those visions, which indicate an impact on HCPs’ work, as relevant to our topic.

##### SCs Transforming the Work of HCPs

Some publications addressed the future role that SCs could play in health care with regard to HCPs and the health care system. SCs might *transform the roles of HCPs,* as they might “overhaul general practice” [34] in terms of patient-centeredness, but AI tailored to GPs should improve performance within the traditional roles of the GP [34]. Some authors expected that *SCs complement HCPs,* as they cannot and will not replace consultations with GPs [43] and face-to-face empathic care [34]. SCs can assist [14,34,41,43] or augment [36] HCPs. Nevertheless, SCs can also be in *competition with HCPs*. They might replace GPs in the context of the tasks that they are able to perform [43]. Moreover, they might play a more significant role in patient health than their HCPs [14].

##### SCs’ Impact on the Health Care System

Some authors addressed the potential impacts of SCs on the health care system. Some assumed the potential for *improved supply*. SCs might help to overcome supply issues associated with inaccessible or unaffordable health care services [45]. Furthermore, they can provide quick and targeted access to appropriate HCPs [41,45]. Other authors were concerned about *potential overuse of services* and the wastage of scarce resources of HCPs. Unnecessary medical visits resulting from the use of SCs might cause unnecessary costs [37].

#### Discursive Patterns About SCs

Looking beyond the respective themes, we identified a spectrum of 4 main discourses in which SCs were discussed as inferior, harmful, beneficial, or superior to humans (Table 3).

In the discourse of *SCs as inferior,* SCs are depicted as deficient in comparison with human physicians or unable to replace human physicians. The discourse of *SCs as harmful* centers the negative effects of SCs on humans such as anxiety and fear among users. The discourse of *SCs as beneficial* contains the positive effects of SCs, for example, for users (knowledge), physicians (relief of repetitive tasks), and the physician-patient relationship in general. In the discourse of *SCs as superior,* they are discussed as potentially outperforming humans or causing effects that are allegedly beyond human reach such as changing the roles of physicians. Thus, our results depict SCs as a double-edged sword in the physicians’ work.

**Table 3:** Symptom checkers (SCs): technology in relation to humans.

Theme, topic, and subtopic				SCs as inferior		SCs as harmful		SCs as beneficial		SCs as superior
**Finding prediagnosis: comparison between SCs and physicians**										
	**Performance of diagnosis**									
		Lower accuracy of SCs in diagnostics and triage	✓		N/A^a^		N/A		N/A	
		Physicians consider various sources of information	✓		N/A		N/A		N/A	
		SCs provide a first sense of urgency	N/A		N/A		✓		N/A	
	**Relevance of the human factor**									
		SCs lack human factor	N/A		N/A		N/A		✓	
		Human factor as a strength of physicians	✓		N/A		N/A		N/A	
**Layperson-technology relationship**										
	**SCs as potential for layperson empowerment**									
		SCs might provide laypersons with knowledge	N/A		N/A		✓		N/A	
		SCs might have layperson activating potential	N/A		N/A		✓		N/A	
	**SCs as potential harm for laypersons**									
		SCs' quality and liability still unclear	✓		N/A		N/A		N/A	
		SCs might increase anxieties in laypersons	N/A		✓		N/A		N/A	
**(Impacts on) physician-patient relationship**										
	**Disruptions of the physician-patient relationship**									
		Potential of changing roles	N/A		N/A		N/A		✓	
	**Uncontested role of HCPs^b^**									
		Improvement of relationship	N/A		N/A		✓		N/A	
		HCPs remain the primary source of trustworthy information	✓		N/A		N/A		N/A	
**Impacts on HCPs’ tasks**										
	**SCs reducing HCPs’ workload**									
		SCs could support repetitive and less-complicated tasks	N/A		N/A		✓		N/A	
		SCs could enable physicians to concentrate on other tasks	N/A		N/A		✓		N/A	
	**SCs increasing HCPs’ workload**									
		SCs might cause unnecessary contacts	N/A		✓		N/A		N/A	
		SCs might cause additional tasks during consultation	N/A		✓		N/A		N/A	
**Future roles of SCs in health care**										
	**SCs transforming the work of HCPs**									
		SCs transform the roles of HCPs	N/A		N/A		N/A		✓	
		SCs complement HCPs	N/A		N/A		✓		N/A	
		SCs in competition with HCPs	N/A		✓		N/A		N/A	
	**SCs' impacts on the health care system**									
		Improved supply for patients	N/A		N/A		✓		N/A	
		Potential overuse of service through patients	N/A		✓		N/A		N/A	

^a^N/A: not applicable.

^b^HCP: health care professional.

## Discussion

### Search Results

#### Main Themes and Types of Literature

SCs are designed for laypersons but might have direct and indirect impacts on HCPs, as patients may use SCs instead of, before, or in addition to the consultation with an HCP. We conducted a scoping review to identify existing knowledge about the impacts of SCs on HCPs in primary care, especially with regard to work content and organization, HCP-patient or lay interaction or relationship, professionals’ identity, job satisfaction, and perceived stress of HCPs. We derived these perspectives from established models of work-related stress and mental health in the workplace [16,17].

We included 15 publications and identified 5 main themes concerning the impacts of SCs on physicians in general practice and other settings. The literature in our scoping review consists of research publications, literature reviews, and nonresearch publications such as commentaries. We found the main themes across all 3 types of literature, with clear differences in the levels of the subtopics. Most subtopics in the themes *layperson-technology relationship* and *(impacts on) physician-patient relationship* were not addressed by research publications, whereas almost all subtopics were covered in literature reviews. This implies that there is a scientific discourse about these topics and that our search strategy was able to find it. Consequently, we assume that there is a lack of empirical knowledge about the experiences of and impacts on laypersons and physicians regarding SCs. In the few research publications included, the physicians expressed expectations about SCs [14,35] rather than lived experiences with SCs [40] in daily routines.

Interestingly, we could see that SCs entering the field of (pre)diagnosis was not enlarged from the perspective of “professionals’ identity,” “job satisfaction,” and “perceived work-related stress” that were further perspectives on our research question. Subtopics such as *SCs might cause unnecessary contacts* or *SCs might cause additional tasks during consultation* might be potential indicators of perceived stress. Subtopics such as *potential of changing roles* in the physician-patient relationship as well as *SCs in competition with HCPs* and *SCs transform the roles of HCPs* in health care might affect HCPs’ professional identities. The reason that these dimensions are addressed rather implicitly might be due to the limited lived experience of HCPs with SCs in the daily routines mentioned in the previous paragraph and the lack of the perspective of occupational health in the context of SCs in health care.

#### Challenges of Heterogeneous Concepts and Wording

The existing literature turned out to be heterogeneous and inconsistent with regard to the concepts and wording that are used (Table 2 provides more details). AI, algorithms, chatbots, and SCs are used, among other concepts. This has also been addressed in other publications as “confusion about definition, purposes and potential of AI in medicine” [47,48]. It also mirrors the heterogeneity of technologies that are collected under the umbrella of SCs as well as the heterogeneous foci that authors choose with their wording. With regard to features of SCs, the wording contains terms such as “symptom checkers” [15], “self-diagnosis” [36], “self-triage” [37], “triage advice” [41,49], or “suggested course of action” [36]. Medical wording such as “diagnosis” or “triage” implies that technology puts these in the hands of laypersons or patients, whereas terms such as “symptom checker” or “suggested course of action” depict the technology as a handbook for or feedback to symptoms for them. This indicates an ongoing debate about what SCs are and what they can provide to users. We see the need to refine the wording, as terms such as “diagnosis” and “triage” might be diluted. This might be misleading for layperson users and HCPs in its implications.

### Strengths and Limitations

The strengths and limitations of our scoping review are related to the methods, search process, findings, and quality assessment.

We used the scoping review methodology using the Arksey and O’Malley framework [23,24]. Reporting followed the PRISMA-ScR guidelines [31]. Scoping reviews aspire to systematic, transparent, and replicable reviews [50] and allow the exploration of emerging research fields with broader and more comprehensive objectives [21]. Thus, it is a suitable methodological approach for a rather new field such as SCs. The included publications varied widely in terms of publication type and methodology; therefore, the meta-analysis was not promising. This fits the scope of the scoping review methodology [23].

We faced some challenges during the search process. A lack of common keywords and inconsistent database indexing for the topics “SCs/AI in self-diagnosis” made a reproducible database search difficult. We conceive that despite using comprehensive search terms, some potentially relevant publications could not be found. These challenges lead to a rather complex search string for database searches and an iterative search process involving an additional literature search [21]. We also reported no relevant findings using a reference search strategy. Conducting additional searches in key journals and other sources, we did not find any theoretical considerations on the impacts of SCs concerning our research questions. Our analysis of publications did not cover all sources and topics on SCs, as we did not search for data from relevant blogs, websites, etc for methodological reasons.

In addition, sometimes GPs were only one group of physicians among several included in a study or the background of the physicians was not specified. In both cases, when in doubt, we included a publication that fits other relevant dimensions of our research question. No publication could be found that explicitly addressed the work of practice assistants.

To ensure the quality of the thematic analysis [32], three researchers (EÖ, NR, and CP)—social scientists working in the field of occupational health and health services research—discussed the results subsequently and received additional feedback from an occupational health physician (MAR) and a researcher working in the field of digitalization in health care and SCs (AJW) [21]. The multiperspectivity of the research team fits the requirements of the overarching research interest and enriches the data-analysis process.

### Comparison With Existing Literature

The themes we identified resonated with the wider context of SCs and AI in health care.

#### Finding Prediagnosis and HCPs’ Work Tasks

We identified the first theme as relevant to our research question, as it revolves around the question of who can conduct a core work content of physicians better: trained physicians or SCs. In the wider literature on SCs, this has repeatedly been addressed as a technical question of diagnostic accuracy. With regard to diagnosis performance, the first empirical studies have shown that the quality varied substantially between SCs [49] and that SCs can match up to the diagnostic accuracy of physicians [51] but tend to be risk averse and wrongly categorized rather trivial cases as emergencies [52]. Similar to the discussion around AI in primary care, our scoping review indicates that SCs for self-diagnosis have advantages over humans in some areas while being much less capable in others: “AI can assist in improving efficiency and quality, but is limited by its inability to possess some human characteristics, such as compassion, empathy and the human touch” [7,47]. Visions similar to other AI-based technologies in medicine are voiced that SCs could increase productivity, for example, by reducing time-consuming repetitive tasks for HCPs and making space for those tasks that require a “real doctor” [7,53]. A study included in our scoping review reflects which of GPs’ microtasks within a diagnostic process are integrated into SCs [43]. Nevertheless, the scientific body of literature lacks empirical studies on the direct impacts of SCs on HCPs’ work content.

#### Layperson-Technology Relationship and Physician-Patient Relationship

Patient work is a core work content of HCPs and the impacts of SCs on laypersons and (potential) patients might thus also affect HCPs’ work content and workload [10]. An ambiguous relationship between the use of internet searches for health complaints and perceived low trustworthiness of information from the internet was observed and described [1]. A similar association can be found for SCs; users report being satisfied with the use of SCs but often do not follow the action recommendation. This may lead to the perception that satisfaction is not necessarily bound to appropriate action recommendations but rather to other aspects of the use [15].

The impact of SCs on the physician-patient relationship is prominently addressed in the publications included in our scoping review and centers on the question of whether the roles of physicians and patients might change through SCs. According to a study on the impacts of patients’ use of social media on their interaction with HCPs, discussing health information collected on the web with HCPs led to perceived “tacit opposition” [54]. This interferes with the intended strive from medical paternalism to patient autonomy and processes, such as shared decision-making. Although there is consensus that patients should engage in their own medical decision-making processes, most forms of decision-making still assume that HCPs have knowledge, understanding, and resources that patients do not have [55]. This implies a hierarchical relationship and hinders discussions and decisions on equal terms between patients and physicians. As most patients already use e-sources such as the internet or SCs to inform themselves, further transformation of the patient-physician relationship is crucial to contextualize knowledge collected by patients to aim for a shared disease model as a basis of shared decision-making [1].

#### Future Role of SCs in Health Care

The publications in our scoping review show that SCs are expected to transform the roles of GPs. This is similar to the expected impacts of AI on the roles of HCPs [7]. Transformations are expected with regard to professional practices, patient care, and improvements in medical practice [56]. The introduction of AI is likely to mean that the skills and experience required of health care providers will change [7]. Despite their technological potential, HCPs show little consideration of concrete changes in their practice [56] or the real-life implications of web-based self-diagnosis [57]. A study on physicians in primary care showed that physicians were open to change as long as it helped fulfill their main goal—providing the best care to patients—and kept their role central in the process [56].

With regard to future roles in health care systems, publications on COVID-19–related SCs show that unnecessary in-person visits may be eliminated [13]. Nevertheless, the advice given by poor-quality SCs may lead to unnecessary care access and pressure on health care systems, particularly primary care [49].

### Practical and Research Implications

#### Practical Implications

On the basis of the literature, SCs can partially outsource and improve preliminary diagnoses and enhance diagnostic decision-making. Furthermore, they could become tools for “appropriate triage advice” by the patients themselves [37] and a “first line support for advice and guidance” to laypersons [49]. Especially in the context of the ongoing COVID-19 pandemic, self-triage tools were ascribed the potential to improve triage efficiency and quickly connect patients with the appropriate care venue, preventing unnecessary emergency department and urgent care visits [13]. As the use of SCs by laypersons will potentially increase, GPs need awareness and understanding of patients’ possible SC use [37,58] to reflect their own concerns. It is important that GPs have knowledge about existing SCs to adequately assess the information provided by patients.

SCs are currently not integrated into daily routines; therefore, GPs cannot control patient risks from using SCs for self-diagnosis and evaluation of a suggested course of action [7]. In addition, there might be confusion about the actual purpose of SCs and whether they could actually reduce service load, improve access to care, and help with patients’ needs for information [49]. Currently, physicians remain alone during the transformation process. Training physicians to properly support this transformation process in the patient-physician relationship is the first effective step in the direction of self-determined health care for patients [59].

#### Research Implications

Although the topic of bias reduction on HCPs’ side has been addressed, there is a research gap concerning exacerbating inequalities [47] through SCs. The potential for primary care transformation and better health system performance remains unresearched and focuses mainly on operational benefits and 24-hour patient access [13]. Our previous work showed that work-related demands in general practice are already high [60,61], indicating that new technologies need to fit the needs and routines of HCPs to find acceptance [62]. Real-life implications of SCs, especially with regard to the changes in the daily working routines of GPs and more generally HCPs in primary care, are not well understood [47,57]. Even though the themes of our scoping review touched upon the factors identified in established models of work-related perceived stress such as work content or work organization, we did not find any results concerning HCPs’ job satisfaction or perceived work-related stress explicitly with regard to SCs. Against this background, a subproject of the current research network CHECK.APP [33] focuses on this topic using a qualitative research approach.

### Conclusions

This is the first scoping review that centers on the perspective of occupational health in the context of the potential impacts of SCs on the work of physicians in primary care. A current ethical reflection on SC chatbots in clinical settings indicates potential impacts on the work of HCPs such as impacts on HCPs’ practical wisdom, workload, and motivation at work [63]. Our results integrate into the broader scientific debate on AI-based tools in medicine and the ambiguity and uncertainty about possible adoption, changes in the relationship or workload of HCPs, and the potential for care in general practice [7]. To date, many publications on SCs have depicted expectations and visions for SCs rather than empirical evidence. As such, more empirical research on the experiences and knowledge of GPs is needed to integrate their perspectives into the scientific discourse and the practical development and implementation of SCs.

## Appendix

Multimedia Appendix 1 PRISMA-ScR (Preferred Reporting Items for Systematic Reviews and Meta-Analyses extension for Scoping Reviews) checklist.

Multimedia Appendix 2 Search strings for PubMed and CINAHL.

## Abbreviations

AI: artificial intelligence
GP: general practitioner
HCP: health care professional
MeSH: Medical Subject Headings
PRISMA-ScR: Preferred Reporting Items for Systematic Reviews and Meta-Analyses extension for Scoping Reviews
SC: symptom checker

## Data Availability

The data sets generated and analyzed during this study are available from the corresponding author upon reasonable request.
