# Supplementary material for: Impacts of Symptom Checkers for Laypersons’ Self-diagnosis on Physicians in Primary Care: Scoping Review
Source: J Med Internet Res. 2023 May 29;25:e39219. doi: 10.2196/39219 (PMC10262026; doi:10.2196/39219)
Supplement: Multimedia Appendix 2 [file jmir_v25i1e39219_app2.docx]

## Multimedia appendix: Search strings

###

### Search string for PubMed (Medline)

**Concepts**: CP 1 ‘Apps’; CP 2 ‘e-Health’; CP 3 ‘Self-Diagnosis’

**Search strategy**: CP 1 **AND** CP 2 **AND** CP 3

| CP 1 | ("app"[Title/Abstract] OR "apps"[Title/Abstract] OR "symptom checker*"[Title/Abstract] OR "self-checker*"[Title/Abstract] OR "chatbot*"[Title/Abstract] OR "mobile application*"[Title/Abstract] OR "AI application*"[Title/Abstract] OR "mobile applications"[MeSH Terms]) |
| --- | --- |
| CP 2 | ("mobile health"[Title/Abstract] OR "e-health"[Title/Abstract] OR "ehealth"[Title/Abstract] OR "mhealth"[Title/Abstract] OR "m-health"[Title/Abstract] OR "mobile technolog*"[Title/Abstract] OR "mobile device*"[Title/Abstract] OR "smartphone*"[Title/Abstract] OR "mobile phone*"[Title/Abstract] OR "cell phone*"[Title/Abstract] OR "cellular phone*"[Title/Abstract] OR "smart phone*"[Title/Abstract] OR "artificial intelligence"[Title/Abstract] OR "Artificially intelligent"[Title/Abstract] OR "AI"[Title/Abstract] OR "computer reasoning"[Title/Abstract] OR "computational intelligence*"[Title/Abstract] OR "machine learning"[Title/Abstract] OR "deep learning"[Title/Abstract] OR "algorithm*"[Title/Abstract] OR "telehealth"[Title/Abstract] OR "Dr. Google"[Title/Abstract] OR "Dr Google"[Title/Abstract] OR "telemedicine"[MeSH Terms] OR "cell phone"[MeSH Terms] OR "smartphone"[MeSH Terms] OR "algorithms"[MeSH Terms]) |
| CP 3 | ("diagnos*"[Title/Abstract] OR "self-diagnos*"[Title/Abstract] OR "self-anamnesis"[Title/Abstract] OR "anamnesis"[Title/Abstract] OR "symptom assessment"[Title/Abstract] OR "initial assessment"[Title/Abstract] OR "self-assessment"[Title/Abstract] OR "symptom evaluation"[Title/Abstract] OR "symptom tracking"[Title/Abstract] OR "pre-screening"[Title/Abstract] OR "triage"[Title/Abstract] OR "triages"[Title/Abstract] OR "self-triage"[Title/Abstract] OR "diagnosis, computer assisted"[MeSH Terms] OR "diagnosis, differential"[MeSH Terms] OR "triage"[MeSH Terms] OR "symptom assessment"[MeSH Terms]) |

### Search string for CINAHL

**Concepts**: CP 1 ‘Apps’; CP 2 ‘e-Health’; CP 3 ‘Self-Diagnosis’

**Search strategy**: CP 1 **AND** CP 2 **AND** CP 3

| CP 1 | MH "Mobile Applications" OR TI app OR AB app OR TI apps OR AB apps OR ((TX symptom OR TX self) N0 (TI checker* OR AB checker*)) OR TI "self-checker" OR AB "self-checker" OR TI chatbot* OR AB chatbot*OR (TX mobile OR TX AI) N2 (TI application* OR AB application*)) |
| --- | --- |
| CP 2 | MH "Cellular Phone" OR MH "Smartphone" OR MH "Decision Making, Computer Assisted" OR MH "Artificial Intelligence+" OR MH "Algorithms" OR MH "Telemedicine+" OR MH "Telehealth" OR MH "Mobile Health Units" OR ((TI mobile OR AB mobile) N0 (TX health OR TX technolog* OR TX device* OR TX phone*)) OR TI ehealth OR AB ehealth OR TI e-health OR AB e-health OR TI mhealth OR AB mhealth OR TI m-health OR AB m-health OR TI smartphone* OR AB smartphone* OR ((TX cell OR TX cellular OR TX smart) N0 (TI phone* OR AB phone*)) OR ((TX artificial OR TX artificially OR TX computational) N0 (TI intelligence OR AB intelligence)) OR TI AI OR AB AI OR ((TX machine OR TX deep) N0 (TI learning OR AB learning)) OR TI algorithm* OR AB algorithm* OR TI telehealth OR AB telehealth OR TI "Dr. Google" OR AB "Dr. Google" OR TI "Dr Google" OR AB "Dr Google" OR TI telemedicine OR AB telemedicine |
| CP 3 | MH "Self Diagnosis" OR MH "Self Assessment" OR MH "Diagnosis, Computer Assisted+" OR MH "Triage" OR MH "Diagnosis, Differential" OR TX diagnos* OR TX "self-diagnos*" OR TX anamnesis OR TX "self-anamnesis" OR ((TX symptom OR TX initial OR TX self OR TX self-) N0 TX assessment*) OR TX "symptom evaluation*" OR TX "pre-screening*" OR TX triage OR TX triages OR TX "self-triage*" |
